# Mesenteric perfusion at rest and during feeding in healthy children

**DOI:** 10.3389/fnetp.2026.1739687

**Published:** 2026-05-14

**Authors:** Kathryn E. Tobert, Kathryn Higdon, Theresa A. Mikhailov, John P. Scott, Wendi Redfern, George M. Hoffman

**Affiliations:** 1 Medical College of Wisconsin, Milwaukee, WI, United States; 2 Division of Critical Care, Department of Pediatrics, Medical College of Wisconsin, Milwaukee, WI, United States; 3 Division of Critical Care, Department of Pediatrics, University of Missouri Health Care, Columbia, MO, United States; 4 Pediatric Intensive Care Unit, Children’s Wisconsin, Milwaukee, WI, United States; 5 Division of Pediatric Anesthesiology and Critical Care Medicine, Department of Anesthesiology, Medical College of Wisconsin, Milwaukee, WI, United States

**Keywords:** enteral nutrition, mesenteric perfusion, near infrared spectroscopy, network physiology, pediatrics, regional oxygen delivery

## Abstract

**Background:**

In critical illness, mesenteric organs are at risk for ischemic injury. Near infrared spectroscopy (NIRS) measures an index of regional venous-weighted tissue oxyhemoglobin saturation, an approximation of regional oxygen supply-demand economy. Mesenteric NIRS measures have been utilized in critically ill neonates to monitor for necrotizing enterocolitis but have not been well-described in healthy infants or children. The purpose of this study was to provide preliminary estimates of normal values for mesenteric regional saturations in healthy infants and young children during activities including feeding.

**Methods:**

We enrolled healthy infants and children between 1 month and 6 years for this observational study. Regional oxygen saturation (rSO_2_) measures were obtained from cerebral, renal, and bilateral mesenteric-abdominal regions before, during, and after consuming a meal. Activity over the period of observation was categorized as calm, asleep, eating, post-prandial, active, and anxious states. We analyzed the association between physiologic parameters and clinical state using multilevel models.

**Results:**

Nineteen subjects (age 33.8 ± 20.7 months, weight 13.5 ± 4.7 kg [mean ± SD]) completed the study. Absolute rSO_2_ measures varied between subjects, with state-dependent changes within subjects that were consistent between subjects. Cerebral rSO_2_ varied little across states. Both mesenteric and renal rSO_2_ varied across states with similar absolute values and trends across 5 of 6 clinical states, differing only in the asleep state, in which only renal rSO_2_ increased. Small decreases in renal (−1.5%, SE 0.7, p < 0.01) and mesenteric (−1.7%, SE 0.7, p < 0.05) rSO_2_ were observed with eating. Larger decreases were observed with anxiety for both renal (−7.7%, SE 1.0, p < 0.001) and mesenteric (−10.3, SE 1.4, p < 0.001) rSO_2_.

**Conclusion:**

In this study of healthy infants and young children, we observed small reductions in mesenteric rSO_2_ in the post-prandial state. In contrast to cerebral rSO_2_, both renal and mesenteric rSO_2_ were significantly influenced by state, particularly activity and anxiety. The state-dependence of regional oxygen economy should be considered when interpreting renal and mesenteric rSO_2_ measures.

## Introduction

All systemic organs have parallel arterial blood supply and thus compete with each other for blood flow. The renal, mesenteric, and hepatic circulations compete with each other and have resistance modulated by local and systemic factors as part of regional and whole-body integrated physiologic network responses. In critical illness, these responses place mesenteric organs at risk for ischemic injury due to inadequate oxygen delivery (DO_2_) (Woolsey and Coopersmith, 2006). Previously established methods of monitoring mesenteric perfusion are either invasive, such as gastric tonometry (Kaufman et al., 2008), or limited in clinical utility due to high cost, technical challenges, or intermittent availability, as in superior mesenteric artery velocity measurement (Gillam-Krakauer et al., 2013). Thus, a non-invasive, cost-effective method to continuously monitor mesenteric perfusion is needed.

Monitoring mesenteric regional tissue oxyhemoglobin saturation *via* near infrared spectroscopy (NIRS) has been used in critically ill neonates to monitor for early signs of necrotizing enterocolitis and risk of feeding intolerance (Schat et al., 2019; Corvaglia et al., 2017; Fortune et al., 2001; Patel et al., 2014). Continuous-wave NIRS devices estimate tissue oxygen saturation values (rSO_2_), using algorithms dependent on sensor geometry and models of tissue capillary oxygen saturation domains bounded by arterial and venous points. The measurement is venous-weighted, as 75%–90% of tissue blood is post-arteriolar, and thus can be used to estimate regional venous oxygen saturation and regional oxygen economy (Martini and Corvaglia, 2018; Scott and Hoffman, 2014; Boushel et al., 2001; Watzman et al., 2000; Nagdyman et al., 2005; Nagdyman et al., 2008).

Previous studies examining the utility of mesenteric NIRS in conditions including anemia, post blood transfusion, and congenital heart disease, have been limited to premature neonates or critically ill infants (Kaufman et al., 2008; Braski et al., 2018; Balegar et al., 2020). Multiple studies have demonstrated age-dependent variation in regional oxygen saturations during first few weeks of life, limiting the extrapolation of mesenteric NIRS trends to older populations (Kuik et al., 2019; McNeill et al., 2011). Normal values and variations for mesenteric rSO_2_ are poorly characterized in healthy infants or young children (McNeill et al., 2011). The introduction of early enteral nutrition in critically ill children may be associated with improved outcomes, but mesenteric ischemia is a significant concern when introducing enteral feeding in this population (Mikhailov et al., 2014). Therefore, reference ranges and changes with feeding for mesenteric regional saturations in healthy infants and children would be helpful to inform further studies in critically ill or injured infants and children.

The purpose of this study was to determine preliminary estimates for normal values for mesenteric regional saturations in healthy infants and young children and to determine whether ingestion of food itself alters NIRS indices of mesenteric oxygen economy. We hypothesized that circulatory control networks would adjust regional blood flow to the demands of enteral feeding in health, and that enteral feeding would not systematically lower mesenteric oxygen balance in this population.

## Materials and methods

### Study approval and design

We obtained approval for the study from the Children’s Wisconsin and Medical College of Wisconsin Institutional Review Board. We conducted a single-center prospective observational study, performed in the outpatient Translational Research Unit, seeking to enroll healthy infant and child volunteers from 1 to 72 months post-term age with a weight limit of 20 kg. These age limits were chosen to avoid circulatory complexity in transitional circulation, and to allow adequate interrogation of intra-abdominal organs with existing path-length constraints (Scott and Hoffman, 2014; Boushel et al., 2001; Balegar et al., 2020).

### Exclusion criteria

We screened healthy volunteers for gastrointestinal disorders, (including chronic constipation, medically treated gastroesophageal reflux, and motility disorders), history of significant abdominal surgeries involving bowel resection or repositioning, cardiovascular abnormalities, respiratory conditions such as asthma that would require treatment during the study period, and dermatologic conditions such as scarring or eczema that would interfere with NIRS probe placement. We asked participants that had experienced any acute symptoms of fever, vomiting, or diarrhea in the 24 h prior to their scheduled study appointment to reschedule.

### Sample size

To determine the needed sample size, we chose a clinically significant 10-point change in mesenteric NIRS with a standard deviation of 10, using a two-sample t-test with 80% power and false-positive (two-sided alpha) rate of 0.05, the estimated a sample size was N = 16. This number was inflated to 20 to allow for non-Gaussian parameter distributions and enrollment attrition.

#### Study period

We monitored study participants for 6 h, during which participants were told to plan an enteral meal. We timed appointments such that approximately half-way through the study period, participants consumed an enteral meal. This allowed for the establishment of a baseline pre-prandial mesenteric rSO_2_ prior to consuming a meal, monitoring of changes that occur during a meal, and evaluation of post-prandial changes as the enteral meal transitions from the stomach to the small intestine. If the participant was a breastfed or bottle fed infant, they were allowed to feed per their normal schedule. Throughout the study period, we asked parents to keep an activity log for the child; we specifically asked parents to make note of when the child ate or drank, used the restroom, had a dirty diaper, or if they became particularly upset or agitated. From these activity logs, we categorized subject activity into six clinical states as asleep, calm, eating, post-prandial, active, and anxious.

### Data collection

During the entire study period, participants wore a finger or toe pulse oximeter probe and four NIRS probes over cerebral, renal, and two mesenteric tissue beds. The cerebral probe was placed on the forehead without specific regard for laterality. The renal probe was placed below the 12th rib on the left side in lateral orientation. Mesenteric NIRS probes were positioned in horizontal orientation over the mid-abdomen on both sides at the level of the umbilicus. Monitored physiologic parameters included heart rate (HR) and arterial oxygen saturation (SpO_2_) from pulse oximeter signals (Masimo SET algorithm, B850 multiparameter monitor, GE Healthcare, Waukesha, WI), cerebral regional oxygen saturation (rSO_2_C), renal regional oxygen saturation (rSO_2_R), right-sided mesenteric regional oxygen saturation (rSO_2_MesR), and left-sided mesenteric regional oxygen saturation (rSO_2_MesL) continuously during the study period. Regional oximetric rSO_2_ measures were obtained from a multichannel continuous-wave NIRS device (neonatal sensors, neonatal/pediatric algorithm, Somanetics INVOS 5100C, Troy, MI; now Medtronic Inc., Minneapolis, MN). All physiologic measures were collected synchronously at 10-s intervals with BedMasterEx (Excel Medical, Jupiter FL; now Hillrom-Welch Allyn, Baxter Inc., Deerfield, IL) and stored at 1-min intervals for subsequent analysis (Liddle et al., 2012). From synchronous measures, derived parameters included a single measure for mesenteric rSO_2_M from the average of left- (rSO_2_MesL) and right- (rSO_2_MesR) sided mesenteric signals, arterial-regional differences including ΔarSO_2_C = SpO_2_ – rSO_2_C, ΔarSO_2_R = SpO_2_ – rSO_2_R, ΔarSO_2_M = SpO_2_ – rSO_2_M, and comparative regional differences including ΔrSO_2_RC = rSO_2_R–rSO_2_C and ΔrSO_2_MC = rSO_2_M–rSO_2_C (Hoffman et al., 2017).

### Statistical analysis

Demographic and physiologic measures were summarized as number and (percent), mean ± standard deviation, or median (interquartile range), and [5%–95% confidence intervals or minimum-maximum]. Multilevel panel regression models with random effects for age, gender, weight, and state were used to assess associations with physiologic measure, with variance correction for clustering within subjects and uncorrected p < 0.05 as significant. Differences between states were tested using fixed effects models, with the calm-awake state as a reference, with Scheffe correction for multiple comparisons. Parameter estimates from the regression models were expressed as point estimate mean ± SE.

## Results

### Demographics and study conditions

**FIGURE 1:**
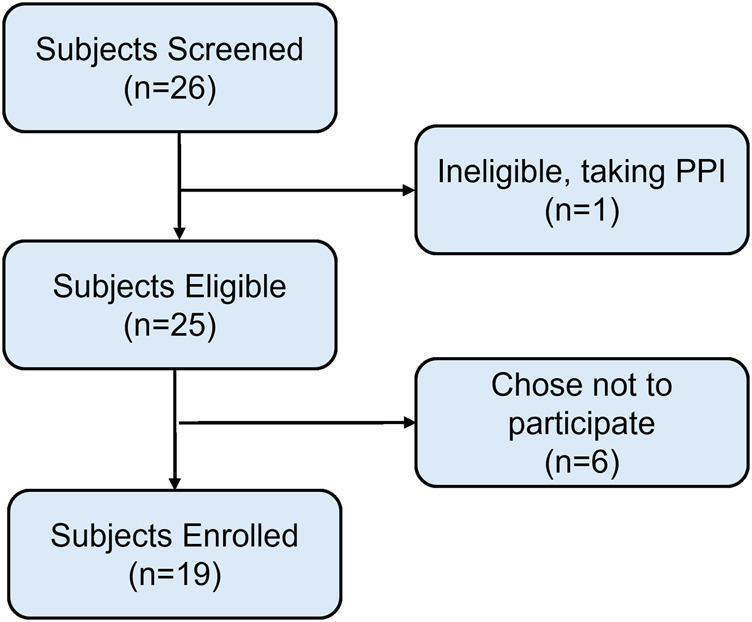
Flow diagram of subject screening and eligibility. In total, six subjects chose not to participate in the study out of the 25 that were eligible after meeting inclusion criteria. PPI, proton-pump inhibitor.

We screened 26 potential participants for eligibility for the study, excluding one subject because of proton-pump inhibitor use. Of 25 eligible participants, 19 were enrolled with parental consent. Familial preference or lack of caregivers for other children were the main reasons that the parents of eligible potential participants ultimately chose not to participate (Figure 1).

The subject population was 11/19 (58%) female, with weight 13.5 ± 4.7 (range 5.5–20.7) kilograms, and age 3 ± 21 (range: 3–68) months. Four patients (21.1%) were <1 year of age, 3 patients (15.8%) were between 1 and 2 years of age, 6 patients (31.6%) were between 3 and 4 years of age, and 6 patients (31.6%) were between 4 and 6 years of age. All participants were able to complete the 6-h trial with all probes in place. Skin irritation was reported after probe removal in one subject.

**FIGURE 2:**
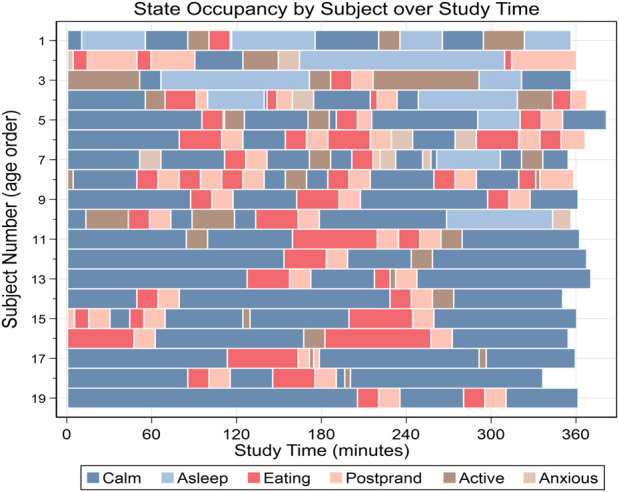
State occupancy progression for subjects over study time.

A range of activities were observed in most subjects, and all were observed in calm, eating, and postprandial states. Table 1 summarizes the time distribution of states observed across subjects. Figure 2 shows the pattern of state occupancy for each subject over their study time.

**TABLE 1:** Distribution of occupancy of observed clinical states across subjects.

State	Occupancy (overall)	Occupancy (between subjects)	Occupancy (within subjects)
Calm	3,814 (56%)	19 (100%)	(56%)
Asleep	707 (10%)	7 (37%)	(28%)
Eating	977 (14%)	19 (100%)	(14%)
Postprandial	726 (11%)	19 (100%)	(11%)
Active	501 (7%)	16 (84%)	(9%)
Anxious	109 (2%)	5 (27%)	(6%)
Total	6,834 (100%)	85 (100%)	(100%)

Occupancy is shown as total minutes and (percent of total). Between-subject occupancy shows the number of subjects observed in each state. Within-subject occupancy shows the average percent of time in occupied states for each subject.

### Regional oxygen saturations across clinical states

Physiologic data were obtained for 6,834 total minutes, with an average of 360 (range 336–381) minutes per subject. We did not use 52 min of observation from 12 subjects because of probable artifact with recorded SpO_2_ < 88%, yielding 6,782 min of data for analysis. Because rSO_2_ measures do not require pulse detection, motion is not a significant source of artifact. Table 2 shows a summary of the observed unweighted physiologic measures across clinical states.

**TABLE 2:** Physiologic measures across clinical states.

Parameter/State	Calm	Asleep	Eating	Postprandial	Active	Anxious	Total
N (%)	3,784 (56)	706 (10)	969 (14)	724 (11)	490 (7)	109 (2)	6,782 (100)
SpO_2_ (%)	99 (1) [88; 100]	98 (2) [94; 100]	99 (1) [88; 100]	99 (1) [90; 100]	99 (2) [89; 100]	99 (1) [95; 100]	99 (2) [88; 100]
HR (bpm)	110 (24) [35; 185]	123 (23) [64; 193]	116 (25) [66; 191]	126 (26) [67; 186]	127 (37) [80; 192]	144 (23) [97; 202]	115 (27) [35; 202]
rSO_2_C (%)	76 (6) [59; 89]	69 (8) [53; 87]	75 (5) [57; 88]	76 (7) [31; 95]	74 (9) [57; 92]	73 (8) [61; 87]	75 (6) [31; 95]
rSO_2_R (%)	88 (9) [56; 95]	93 (7) [67; 95]	86 (10) [27; 95]	87 (10) [54; 95]	86 (9) [61; 95]	82 (14) [55; 95]	87 (11) [27; 95]
rSO_2_MesR (%)	87 (11) [41; 95]	80 (15) [54; 95]	84 (18) [45; 95]	81 (14) [31; 95]	86 (6) [58; 95]	70 (15) [36; 95]	85 (13) [31; 95]
rSO_2_MesL (%)	86 (15) [36; 95]	83 (11) [62; 95]	84 (20) [29; 95]	81 (16) [49; 95]	82 (10) [50; 95]	70 (19) [40; 90]	84 (15) [29; 95]
rSO_2_M (%)	86 (12) [42; 95]	79 (8) [60; 94]	84 (18) [51; 95]	81 (14) [44; 95]	84 (8) [64; 95]	68 (12) [42; 88]	84 (14) [42; 95]
ΔarSO_2_C (%)	23 (6) [7; 41]	29 (8) [12; 44]	24 (6) [10; 42]	23 (8) [5; 68]	24 (8) [6; 43]	26 (9) [11; 39]	24 (7) [5; 68]
ΔarSO_2_R (%)	12 (9) [0; 43]	6 (8) [0; 33]	13 (10) [0; 71]	13 (10) [2; 44]	12 (8) [0; 37]	17 (14) [5; 45]	12 (11) [0; 71]
ΔarSO_2_MesR (%)	12 (11) [0; 58]	17 (15) [2; 45]	15 (19) [0; 52]	18 (12) [2; 68]	12 (7) [0; 41]	29 (14) [12; 63]	14 (13) [0; 68]
ΔarSO_2_MesL (%)	13 (15) [0; 63]	15 (11) [3; 37]	16 (20) [0; 70]	18 (16) [2; 50]	16 (10) [0; 50]	29 (19) [10; 60]	15 (15) [0; 70]
ΔarSO_2_M (%)	14 (12) [0; 56]	19 (8) [4; 40]	16 (18) [0; 49]	18 (13) [2; 55]	14 (8) [2; 36]	30 (12) [12; 58]	15 (13) [0; 58]
ΔrSO_2_RC (%)	11 (9) [−25; 32]	22 (8) [−5; 38]	10 (10) [−54; 32]	9 (11) [−12; 43]	11 (11) [−15; 32]	7 (16) [−16; 32]	12 (10) [−54; 43]
ΔrSO_2_MC (%)	10 (12) [−42; 34]	11 (11) [−12; 32]	8 (10) [−29; 31]	4 (15) [−32; 39]	10 (10) [−15; 32]	;2 (15) [−41; 16]	10 (11) [−42; 39]
ΔrSO_2_RM (%)	2 (8) [−20; 32]	10 (12) [−11; 28]	0 (12) [−54; 33]	4 (12) [−19; 30]	2 (8) [−21; 19]	12 (14) [−16; 10]	2 (10) [−54; 33]

Values are unweighted median (IQR) and [range] of all observations across all subjects and states.

Abbreviations: SpO_2_, arterial oxygen saturation; HR, heart rate; rSO_2_C, cerebral regional oxygen saturation; rSO_2_R, renal regional oxygen saturation; rSO_2_MesR, right-sided mesenteric regional oxygen saturation; and rSO_2_MesL, left-sided mesenteric regional oxygen saturation, rSO_2_M from the average rSO_2_MesL and rSO_2_MesR, ΔarSO_2_C = SpO_2_ – rSO_2_C, ΔarSO_2_R = SpO_2_ – rSO_2_R, ΔarSO_2_MesR = SpO_2_ – rSO_2_MesR, ΔarSO_2_MesL = SpO_2_ – rSO_2_MesL, ΔarSO_2_M = SpO_2_ – rSO_2_M, ΔrSO_2_RC = rSO_2_R–rSO_2_C, ΔrSO_2_MC = rSO_2_M–rSO_2_C, and ΔrSO_2_RM = rSO_2_R–rSO_2_M.

The associations of patient characteristics and activity with physiologic parameters in random-effects models are shown in Table 3. Heart rate was positively associated with male sex and negatively associated with weight. Mesenteric rSO_2_ was positively associated with age. No other measures showed significant associations with age, weight, or sex.

**TABLE 3:** Random effects models of physiologic measures.

Parameter/Model	HR	SpO_2_	rSO_2_C	rSO_2_R	rSO_2_MesR	rSO_2_MesL	rSO_2_M
Age	0.0203 (0.302)	0.00771 (0.016)	0.0848 (0.148)	0.302 (0.159)	0.499** (0.155)	0.397 (0.297)	0.449* (0.210)
Weight	−3.765** (1.386)	−0.0132 (0.063)	0.184 (0.609)	−0.832 (0.63)	−1.285 (0.775)	−0.692 (1.247)	−0.992 (0.906)
Sex (male)	13.38** (4.609)	−0.186 (0.289)	2.404 (2.064)	2.054 (2.701)	4.827 (3.24)	4.591 (3.68)	4.725 (2.933)
Activity calm (reference)	0	0	0	0	0	0	0
Asleep	−17.22*** (2.166)	−0.966*** (0.248)	−1.581 (1.56)	5.725*** (1.206)	−1.51 (2.198)	0.66 (1.395)	−0.423 (1.428)
Eating	4.625*** (0.981)	0.0753 (0.068)	−0.464 (0.347)	−1.773** (0.624)	−1.800** (0.636)	−1.908* (0.853)	−1.847** (0.659)
Postprandial	2.981 (1.947)	0.0841 (0.067)	0.316 (0.602	−1.389 (0.92)	−1.122 (1.224)	−2.054** (0.666)	−1.587 (0.886)
Active	2.512 (1.951)	−0.218 (0.229)	0.759 (0.625)	−0.306 (0.882)	0.395 (1.196)	−1.015 (1.608)	−0.305 (1.335)
Anxious	11.09*** (2.741)	−0.258 (0.208)	0.211 (0.857)	−7.787*** (1.00)	−9.080*** (1.54)	−9.785*** (1.154)	−9.416*** (1.276)
Constan	161.0*** (9.078)	99.03*** (0.362)	68.47*** (3.561)	86.53*** (5.148)	82.57*** (5.138)	77.18*** (6.79)	79.88*** (4.995)
Observations	5,309	6,678	6,615	6,612	6,626	6,645	6,650
Sigma_u	8.477	0.464	3.176	4.888	4.768	6.279	4.786
Sigma_e	10.57	1.143	3.472	4.773	6.258	6.322	5.466

Values are coefficient point estimate mean and (se); * p < 0.05; ** p < 0.01; *** p < 0.001. Sigma_u, standard deviation of between-subjects parameter; Sigma_e, standard deviation of residual error term (unmeasured variation).

Absolute regional oxygen saturations varied between subjects, with state-dependent changes within subjects that were consistent between subjects. Small associations were observed for SpO_2_ and rSO_2_C with activity states. Large negative associations for rSO_2_R and all mesenteric rSO_2_M measures were seen with the anxious state, and small negative associations with the eating and post-prandial states. A relatively large positive association was observed between sleep and rSO_2_R.

**FIGURE 3:**
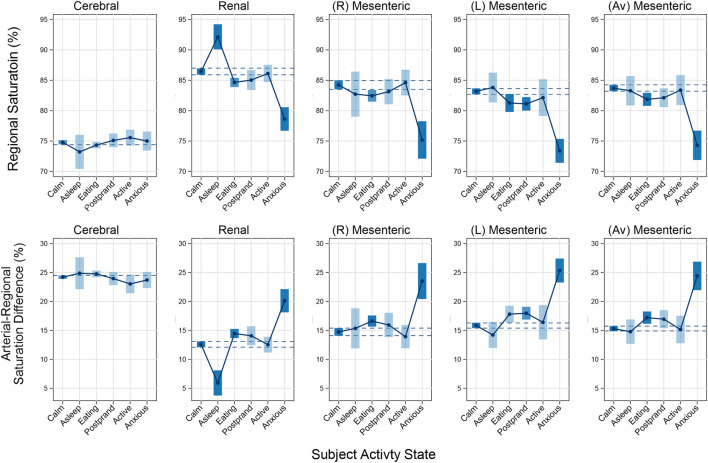
Graphical display of regional saturations (top panel) and arterial-regional saturation differences (bottom panel) across clinical states. Data are point estimates (circle) and 95% CI (bars) for parameters from fixed effects models in Table 4. The reference (calm) state is leftmost in each panel, with 95% CI interval depicted by dashed lines across each state. Significant differences from the reference state are depicted by heavily shaded bars.

Fixed-effect models were constructed to emphasize within-subject state-related changes, shown graphically in Figure 3, and detailed in Table 4. Cerebral regional oxygen saturations showed little variation across all activity states and did not demonstrate any clinically significant differences in any of the states compared to the reference calm, awake state.

**TABLE 4:** Fixed-effect model: changes over activity states.

Parameter/State	Calm	Asleep	Eating	Postprandial	Active	Anxious
HR	118.5 (0.209)	101.3*** (0.52)	123.1*** (0.384)	121.5 (0.444)	121.0 (0.606)	129.6*** (1.077)
SpO_2_	99.04 (0.020)	98.08*** (0.052)	99.12 (0.038)	99.13 (0.441)	98.82 (0.057)	98.78*** (0.117)
rSO_2_C	74.80 (0.061)	73.23 (0.156)	74.33 (0.115)	75.11 (0.134)	75.56 (0.172)	75.00 (0.36)
rSO_2_R	86.43 (0.049)	92.15*** (0.213)	84.65** (0.158)	85.04 (0.184)	86.12 (0.236)	78.63*** (0.49)
rSO_2_MesR	84.25 (0.111)	82.72 (0.28)	82.45** (0.208)	83.14 (0.241)	84.61 (0.309)	75.19*** (0.642)
rSO_2_MesL	83.18 (0.112)	83.81 (0.283)	81.27* (0.21)	81.12** (0.243)	82.14 (0.311)	73.38*** (0.649)
rSO_2_M	83.72 (0.097)	83.27 (0.244)	81.87** (0.181)	82.13 (0.21)	83.38 (0.269)	74.30*** (0.561)
ΔarSO_2_C	24.19 (0.066)	24.88 (0.166)	24.76 (0.123)	23.96 (0.143)	23.03 (0.186)	23.71 (0.381)
ΔarSO_2_R	12.60 (0.086)	5.925*** (0.217)	14.47** (0.16)	14.08 (0.186)	12.54 (0.243)	20.13*** (0.494)
ΔarSO_2_MesR	14.75 (0.111)	15.36 (0.28)	16.62** (0.206)	15.94 (0.24)	13.92 (0.313)	23.52*** (0.636)
ΔarSO_2_MesL	15.86 (0.113)	14.22 (0.285)	17.81* (0.21)	17.97** (0.244)	16.40 (0.318)	25.35*** (0.649)
ΔarSO_2_M	15.30 (0.097)	14.78 (0.245)	17.21** (0.181)	16.95 (0.21)	15.16 (0.273)	24.42*** (0.558)
ΔrSO_2_RC (%)	11.62 (0.289)	18.94*** (1.367)	10.29 (9.877)	9.87 (0.693)	10.58 (1.253)	3.463*** (1.376)
ΔrSO_2_MC (%)	8.93 (0.224)	10.11 (1.619)	7.53 (0.602)	7.02 (0.924)	7.88 (1.771)	−0.85*** (1.814)
ΔrSO_2_RM (%)	2.68 (0.291)	8.83** (1.370)	2.73 (0.634)	2.87 (0.630)	2.68 (0.956)	4.29 (1.228)

All parameter models showed overall significant differences across states at p < 0.001. Differences compared to calm (reference) state: * p < 0.05; ** p < 0.01; *** p < 0.001.

Abbreviations: SpO_2,_ arterial oxygen saturation; HR, heart rate; rSO_2_C, cerebral regional oxygen saturation; rSO_2_R, renal regional oxygen saturation; rSO_2_MesR, right-sided mesenteric regional oxygen saturation; and rSO_2_MesL, left-sided mesenteric regional oxygen saturation, rSO_2_M from the average rSO_2_MesL and rSO_2_MesR, ΔarSO_2_C = SpO_2_ – rSO_2_C, ΔarSO_2_R = SpO_2_ – rSO_2_R, ΔarSO_2_MesR = SpO_2_ – rSO_2_MesR, ΔarSO_2_MesL = SpO_2_ – rSO_2_MesL, ΔarSO_2_M = SpO_2_ – rSO_2_M, ΔrSO_2_RC = rSO_2_R–rSO_2_C, ΔrSO_2_MC = rSO_2_M–rSO_2_C, and ΔrSO_2_RM = rSO_2_R–rSO_2_M.

Renal and mesenteric rSO_2_ were similar both in absolute values and trends across 5 of 6 states, with small decreases during eating and postprandial states, and larger decreases with the anxious state. The renal and mesenteric patterns differed only in the asleep state, during which renal rSO_2_R increased from 86.4% to 92.2% (difference +5.6%, SE 1.4, p < 0.001), with no significant change in any mesenteric measure.

The anxious state produced the largest and most consistently significant changes in both renal and mesenteric NIRS measures. Renal rSO_2_ decreased from 86.4% in the calm state to 78.6% in the anxious state (difference −7.7%, SE 1.0, p < 0.001). Right-sided mesenteric rSO_2_ decreased from 83.3% to 75.2% (difference −8.3%, SE 1.7, p < 0.001). Left-sided mesenteric rSO_2_ decreased from 83.2% to 73.4% (difference −9.7%, SE 1.1, p < 0.001). Combined mesenteric rSO_2_ decreased from 83.8% to 74.3% (difference −9.3%, SE 1.1, p < 0.001).

Small changes were also observed in renal and mesenteric rSO_2_ in the eating and post-prandial states. Most notably, small decreases in renal and bilateral mesenteric rSO_2_ were seen while eating. Renal rSO_2_ decreased to an average of 84.5% in the eating state (difference −1.5%, SE 0.7, p < 0.01). Right- and left-sided mesenteric rSO_2_ decreased to 81.5% and 81.2% respectively in the eating state (difference −1.7% and −1.7%, SE 0.7 and 0.9, p < 0.05 for both). Additionally, left-sided mesenteric rSO_2_ showed a statistically significant decrease to an average of 80.9% in the post-prandial state (difference −2.0%, SE 0.7, p < 0.01).

## Discussion

In this prospective observational study, we performed multi-site rSO_2_ monitoring of regional circulations with NIRS in healthy infants and young children and established preliminary estimates for normal mesenteric rSO_2_ values. Importantly, we categorized clinical states and demonstrated differences in NIRS measures across clinical states and between organ-specific sites. This study of the complex dynamic interactions between the function of feeding and the physiological responses to feeding in healthy children utilized continuous data collection from non-invasive monitors of tissue oxyhemoglobin saturation over a 6-h time-period to demonstrate differences in NIRS measures across clinical states and between organ-specific sites. This demonstrated the importance of understanding network physiology in clinical practice in the care of children.

Cerebral rSO_2_ showed little change across all six clinical states, which is consistent with functional cerebral pressure autoregulation in healthy subjects (Lassen, 1959). Mesenteric and renal rSO_2_ values were largely similar both in absolute values and trends across different clinical states. Both were influenced by clinical state, particularly the anxious state. Both renal and mesenteric circulations have intense sympathetic innervation, and the changes in NIRS measures in those circulations can be partly explained by changes in sympathetic activation. However, renal rSO_2_ increased significantly while sleeping, while mesenteric rSO_2_ did not change significantly. The significant effects of anxiety and feeding on mesenteric measures may suggest a physiologic substrate for the complex interactions between emotional state and feeding behaviors. The regional difference measures (ΔrSO_2_XX deltas, Table 4) highlight differences in patterns of responses between regions.

Because SpO_2_ was minimally variable in this study, the changes in arterio-regional oxygen saturation differences (ΔdarSO_2_X deltas, Table 4) nearly identically mirrored the changes in regional rSO_2_. The small changes in SpO_2_ during sleep were likely clinically insignificant in this healthy young population but may be numerically and clinically important in other conditions and populations, especially those with cyanotic heart disease. In those populations, the arterio-regional saturation differences would provide additional signals reflective of changes in tissue oxygen economy.

Regarding the second aim of this study, to examine changes in mesenteric rSO_2_ with enteral feeding, we observed a small, statistically significant decrease in the bilateral mesenteric and renal rSO_2_ in the eating clinical state as well as a small, statistically significant decrease in the left-sided mesenteric rSO_2_ in the post-prandial clinical state. These observations could reflect a subtle change in the mesenteric oxygen supply and demand relationship in the abdominal organs involved in digestion that requires further investigation.

Assignment of clinical state to a free range of activity was informed by parental activity logs and necessarily collapse continuously variable activity and emotions to artificial categorical descriptors. This was done as an approach to characterize state- and time-dependent variation in physiology. Time-dependent changes within states were not examined in this study. Other sources of unmeasured between-subject variation such as skin pigmentation were not measured in this study. We emphasize that measured and unmeasured sources of variation between subjects are not important for fixed-effect models, which use each subject as their own control across states, as presented in Figure 3 and Table 4.

Previous studies involving neonates have utilized alternative placements for mesenteric NIRS probes. Most studies opted for a midline infraumbilical NIRS probe placement in the small premature infants (Kaufman et al., 2008; Schat et al., 2019; Corvaglia et al., 2017; Fortune et al., 2001; Martini and Corvaglia, 2018; Braski et al., 2018; Balegar et al., 2020; Kuik et al., 2019; McNeill et al., 2011; Corvaglia et al., 2014). Several other studies in premature neonates elected to study right-sided NIRS probe placement (Patel et al., 2014; Cortez et al., 2011). In this study, we placed bilateral NIRS probes at the level of the umbilicus on either side of the umbilicus. We avoided the infraumbilical position to prevent accidently capturing bladder tissue oxygenation. We chose bilateral positioning with the understanding that the right-sided mesenteric NIRS probe may pick up hepatic regional oxygen saturation and differences between the two probes may indicate a change in these regional saturations in healthy children during enteral feeding. However, we saw limited significant differences between the two mesenteric probes, with the only statistically significant change between probes observed in the post-prandial clinical state.

Very little previous literature discusses the frequency and severity of adverse events when using NIRS probes. Skin excoriation and bruising has previously been described in premature infants at less than 48 h of age (Cortez et al., 2011). Out of 19 subjects that have completed the study, one incidence of skin change was reported. There was no report of pruritis, or pain associated with this mild rash. The rash disappeared over the course of several days with no lasting skin effects.

This study has several limitations. Most importantly, enrollment was limited due to obstacles in the early part of the COVID-19 pandemic. Fortunately, the translational research facility at our center remained open throughout the study, but visitor regulations were much stricter. Several COVID-19 related restrictions directly affected our study enrollment including participants or their parent testing positive for COVID-19 in the 2 weeks before their scheduled appointment and visitor limitations preventing siblings from attending appointment with the participants and necessitating those parents find alternative childcare arrangements for siblings. We also anticipate that multiple parents were uncomfortable bringing their healthy children into a hospital during a pandemic, further limiting the number of volunteers. Additionally, although the range in age and size of subjects was wide, developmental changes in regional hemodynamics likely contributed to variation in baseline measures and changes across states. We therefore encourage interpretation of the findings as informative of state-related trends rather than absolute values.

All NIRS rSO_2_ measures are limited by lack of absolute precision, because validation methods have relied on a defined arterio-venous proportional contribution to field saturation, and on assumptions about the path of recovered photons through illuminated tissue. Thus, differences in sensor geometry (near and far source-detector spacing) and algorithms will affect the relationship between rSO_2_ recovered from the illuminated field and the regional venous saturation. Therefore, differences in absolute rSO_2_ indices would be expected between measures using different NIRS systems. However, we would expect that the patterns of change from baseline with changes in subject state would be observable with different rSO_2_ algorithms. With these considerations, we think the findings in this report are generalizable and will inform interpretation of NIRS measures in more vulnerable populations.

In conclusion, this study supports the utility of NIRS monitoring of renal and mesenteric circulations in children with critical illness as a tool to assess the oxygen delivery and consumption relationship of different regional somatic tissues. Although renal NIRS monitoring has been previously studied and is more widely accepted as a means of monitoring critically ill children that may experience hemodynamic compromise, to our knowledge this was the first report of mesenteric NIRS in children beyond infancy. Finally, this study provides preliminary reference values for mesenteric rSO_2_ in healthy infants and children (aged 6 and under and less than 20 kg) and state-dependent changes observed with relatively unconstrained changes in activity and food ingestion. These findings can be utilized as reference points for further studies involving critically ill infants and children.

## Data Availability

The raw data supporting the conclusions of this article will be made available by the authors, without undue reservation.
